# Kinetics of antibodies against pneumococcal proteins and their relationship to nasopharyngeal carriage in the first two months of life

**DOI:** 10.1371/journal.pone.0185824

**Published:** 2017-10-05

**Authors:** Awa L. Mendy, Schadrac C. Agbla, Aderonke A. Odutola, Martin Antonio, Brian M. Greenwood, Jayne S. Sutherland, Martin O. C. Ota

**Affiliations:** 1 Vaccines & Immunity Theme, Medical Research Council Unit, Fajara, The Gambia; 2 Faculty of Epidemiology and Population Health, London School of Hygiene & Tropical Medicine, London, United Kingdom; 3 Faculty of Infectious and Tropical Diseases, London School of Hygiene & Tropical Medicine, London, United Kingdom; 4 Division of Microbiology & Immunity, Warwick Medical School, University Of Warwick, Coventry, United Kingdom; 5 Health Information and Knowledge Management, Health Systems and Services Cluster, WHO Regional Office for Africa, Brazzaville, Congo; Instituto Butantan, BRAZIL

## Abstract

**Introduction:**

The currently used *Streptococcus pneumoniae* vaccines have had a significant impact on the pneumococcal diseases caused by the serotypes they cover. Their limitations have stimulated a search for alternate vaccines that will cover all serotypes, be affordable and effective in young children. Pneumococcal protein antigens are potential vaccine candidates that may meet some of the shortfalls of the current vaccines. Thus, this study aimed to determine the relationship between antibodies against pneumococcal protein antigens and nasopharyngeal carriage in infants.

**Methods:**

One hundred and twenty mother-infant pairs were enrolled into the study. They had nasopharyngeal swabs(NPS) taken at birth and every two weeks for the first eight weeks after delivery, and blood samples were obtained at birth and every four weeks for the first eight weeks after delivery. Nasopharyngeal carriage of *S*. *pneumoniae* was determined from the NPS and antibodies against the pneumococcal proteins CbpA, PspA and rPly were measured in the blood samples.

**Results:**

The *S*. *pneumoniae* carriage rate in infants increased to that of mothers by eight weeks of age. The odds of carriage in infants was 6.2 times (95% CI: 2.0–18.9) higher when their mothers were also carriers. Bacterial density in infants was lower at birth compared to their mothers (p = 0.004), but increased with age and became higher than that of their mothers at weeks 4 (p = 0.009), 6 (p = 0.002) and 8 (p<0.0001). At birth, the infants’ antibodies against *CbpA*, and *rPly* pneumococcal protein antigens were similar, but that of *PspA* was lower (p<0.0001), compared to their mothers. Higher antibody concentrations to *CbpA* [OR (95% CI): 0.49 (0.26–0.92, p = 0.03)], but not *PspA* and *rPly*, were associated with protection against carriage in the infants.

**Conclusion:**

Naturally induced antibodies against the three pneumococcal protein antigens were transferred from mother to child. The proportion of infants with nasopharyngeal carriage and the bacterial density of *S*. *pneumoniae* increased with age within the first eight weeks of life. Higher concentrations of antibodies against *CbpA*, but not *PspA* and *rPly*, were associated with reduced risk of nasopharyngeal carriage of *S*. *pneumoniae* in infants.

## Introduction

The recent estimate of the number of deaths from pneumonia in children under five years is believed to be about 900,000 about a half of which is believed to be due to pneumococcal disease, and mainly in developing countries.[1] The causative agent, *Streptococcus pneumoniae* (the pneumococcus), has over 90 serotypes and normally inhabits the nasopharynx, a state that is assumed to be a precursor of disease, as shown by the association between high rates of pneumococcal carriage and incidence of IPD.[2,3]

Currently, there are two types of pneumococcal vaccines, capsular polysaccharide (PS) and polysaccharide conjugate vaccines (PCV). PS vaccines are poorly immunogenic in children below the age of two years, fail at any age to generate immunological memory, and do not provide herd immunity, making them of limited use for protection of young children. In contrast, PCV are immunogenic in young children and protect against IPD as well as carriage.[4,5] Although PCV are effective, their efficacy is limited to the serotypes contained in the vaccines [6] and there is evidence of an increase in the prevalence of carriage and IPD caused by non-vaccine serotypes in populations where PCV have been used widely.[7] The complex chemistry involved has limited attempts to increase the number of serotypes contained in a vaccine formulation. Nonetheless, there are several vaccine formulations with additional serotypes currently under evaluation. Moreover, PCV are expensive to produce and attempts to reduce cost by using alternate, reduced dosing schedules have so far been inconclusive. [8–10]

The limitations of the available pneumococcal vaccines have warranted a search for alternate vaccines, including those based on pneumococcal protein antigens that are conserved across all serotypes and likely to be immunogenic and cheap. A number of such antigens are currently being investigated as potential vaccine candidates including pneumococcal surface protein A (*PspA*), pneumolysin *(Ply)*, and choline-binding protein A (*CbpA*) [11]. Studies in mice have shown that vaccination with these proteins can protect against disease as well as nasopharyngeal carriage of the pneumococcus.[12,13] Vaccination with a single recombinant *PspA* variant in humans elicited broadly cross-reactive antibodies to heterologous *PspA* molecules.[14] In Sweden, it has been shown in a phase 1 trial that two doses of a tri-component PhtD-dPly-PD vaccine was immunogenic in adults, inducing increases in antibody concentrations and antigen-specific Th1- and Th17-directed cell-mediated immune responses after each dose. [15] A single dose of a protein-based pneumococcal vaccine containing polysaccharide conjugates of 10 pneumococcal serotypes combined with pneumolysin toxoid(dPly) and pneumococcal histidine triad protein D(PhtD) (PHiD-CV/dPly/PhtD-30) in 2–4 years old Gambian children, not previously vaccinated against *S*. *pneumoniae*, was well tolerated and immunogenic.[16] However, in the same setting, this vaccine had no impact on pneumococcal nasopharyngeal carriage in infants, regardless of protein dose or schedule.[17]

Demonstration of a correlation between anti-capsular antibody concentration and clinical efficacy has facilitated the evaluation of new PCV. [18–20]. However, investigations of the relationship between antibodies to protein antigens and nasopharyngeal carriage, a first step in the progression to IPD, have yielded contradictory results. [21, 22]. Therefore, this study aimed to evaluate the kinetics of antibodies to three major pneumococcal protein antigens in relation to nasopharyngeal carriage in early infancy before a pneumococcal conjugate vaccine is given. We hypothesized that the risk of nasopharyngeal carriage by the pneumococcus within the first eight weeks of life would be associated with the amount and kinetics of antibodies against the pneumococcal protein antigens transferred to the infants from their mothers.

## Subjects and methods

### Study setting and population

This was a longitudinal, cohort study conducted at two health centres in The Gambia—Fajikunda and Brikama. These are both peri-urban towns in the Western Region, within 20 and 30 km respectively from the capital Banjul, and with a population of approximately 150,000. This area has a long dry season from December to July and a shorter rainy season. Malaria infection is uncommon in this peri-urban area. Immunization coverage in The Gambia for third dose of diphtheria, pertussis and tetanus vaccines (DPT3) has been above 80% for over a decade. The seven-valent PCV (PCV7) was introduced into the Expanded Programme of Immunisation (EPI) schedule of The Gambia in 2009 and replaced by PCV13 in 2011. PCV13 is given along with other childhood vaccines DPT, Hib and OPV at 2, 3 and 4 months of age. The study described in this paper was conducted between 2013 and 2014.

Healthy pregnant women in their third trimester attending the antenatal clinic in either of the two study health centres were invited to join the study. Written informed consent was obtained. Procedures involved in the study, as well as consent, were reconfirmed on presentation at the delivery room.

### Sample collection times

Nasopharyngeal swabs (NPS) were taken from both mother and child in the delivery room, and during follow-up visits at home every two weeks for the first eight weeks of life, coinciding with the period before the infants received their first dose of PCV. 2ml heparinised blood samples were collected from the mother and from the umbilical cord at delivery. Thereafter, blood samples were collected from both mother and baby four and eight weeks after delivery. The blood and NPS samples were transported to the laboratory within six hours of collection. Field workers were trained in the procedure for obtaining NPS and venous blood samples. The study was approved by the joint Gambia Government/MRC Ethics Committee.

### Nasopharyngeal swab collection and isolation of the pneumococcus

NPS were collected as previously described.[23] Briefly, a Dacron-tipped swab (Corsham, UK) was inserted gently through the nostril and left to soak for about 5 seconds in the nasopharynx, rotated gently and removed. After the NPS had been obtained, the tip was excised immediately and placed in a cryovial containing 1ml skim milk-tryptone-glucose-glycerol (STGG) medium, and placed in a cold box with ice-packs. NPS-STGG specimens were then transported within six hours to the laboratory at the MRC Unit at Fajara, and stored at– 70°C until analyzed.

For culture of *S*. *pneumoniae* from the NPS, the specimens were thawed to room temperature, vortexed and 50 microliters of sample plated onto gentamicin supplemented, sheep blood agar (GBA) (BioMerieux, USA) and incubated overnight at 37°C in 5% CO_2_. Morphologically distinct alpha-hemolytic colonies were sub-cultured onto plain sheep blood agar. *S*. *pneumoniae* was identified by colonial morphology and optochin disc susceptibility (Oxoid, UK). Isolates with reduced optochin disc zone diameters (7–13 mm) were confirmed as *S*. *pneumoniae;* pure isolates were stored at -70°C.

For the isolation of *S*. *pneumoniae* by molecular technique, DNA samples were extracted from the thawed NPS-STGG specimens. Extraction was done using a QIAamp DNA Blood Mini kit (Qiagen, Switzerland) according to manufacturer’s instructions. Briefly, 200μl of NPS sample was resuspended in 50μl of digestion buffer (50 mM Tris-HCl [pH 8.5], 1 mM EDTA, 0.5% sodium dodecyl sulfate, 200 mg of proteinase K per ml), the suspension was incubated on a shaker for 1h at 37°C. 100ul AE buffer was then added to elute the DNA, which was stored at -80°C until analysis by RT-PCR.

### Real-time Polymerase Chain Reaction (RT-PCR)

The RT-PCR assay was carried out in a final 25μl reaction volume and was performed using TaqMan Universal Master Mix (Applied Biosystems, USA) with 2.5μl of DNA. The sequence for the real-time primers and probe used were as previously published and derived from the autolysis gene (lytA) of *S*. *pneumoniae* (Forward: 5′-ACG CAA TCT AGC AGA TGA AGC-3′, Reverse: TCG 5′-TGC GTT TTA ATT CCA GCT-3′, probe: 5′-FAM-GCCGAAAACGCTTGATACAGGGAG-3′-BHQ1). [24] The PCR assay was run using the Corbett Rotor-Gene 6000 (Qiagen, Belgium) with the following cycling parameters: 50°C for 2 min, 95°C for 10 min, followed by 45 cycles of 15s at 95°C and 1 min at 60°C. Pneumococcal colonization density was determined through the creation of standard curves using 10-fold serial dilutions of an in-house concentrated DNA from a purified *S*. *pneumoniae* strain with calculation of pathogen density (copies/milliliter) from the sample cycle threshold (Ct) values as previously described. [25] Amplification data were analyzed by instrument software (Rotor-Gene software series 1.7). Cycle threshold (Ct) values≤40 and >40 were considered positive and negative respectively.

### Immunoassay for antibodies against pneumococcal protein antigens

Serum was separated from venous and cord blood samples and stored at -20°C until analyzed. Antibodies against *PspA*, *rPly* and *CbpA* were measured using in-house enzyme-linked immunosorbent assays (ELISAs). Ninety-six well microtiter plates (Maxisorp Nunc, Denmark) were individually coated with 50μl/well of 5μg/ml for *CbpA*, *PspA*, and *rPly* (all kindly provided by Prof. James Paton, Adelaide University, Australia) in phosphate-buffered saline (PBS) and incubated overnight at 4°C. Plates were then washed twice with PBS Tween 20 (Sigma, USA) and blocked with 10% Fetal Bovine Serum (FBS) in PBS for 1h at 37°C. Subgam (Bio Products Laboratory Limited, UK) which contains known concentrations in mg/ml of IgG antibody titers to *Ply*, *PspA*, and *CbpA*, was used as the standard, starting with a 1/500 dilution that was assigned an antibody titer of 2000ug/ml, for each of the protein antigens. Serial two-fold dilutions of the standard were made in blocking buffer. Serum samples were diluted 1:8 in blocking buffer (10% FBS), and 50μl/well of serum samples and standards were added to appropriate wells in duplicate and incubated for 90min. at 150 rpm at room temperature. Following a wash step, anti-human IgG alkaline phosphatase conjugate diluted 1:2000 in blocking buffer was added and incubated for a further 90 mins. After washing, colour was developed by adding 50ul per well of enzyme reaction substrate *p*-Nitrophenyl Phosphate (pNPP sigma, USA) and incubated in the dark at room temperature for a final 30mins. The plate was then read at 405nm on a Spectramax plate reader with Softmax Pro software (Molecular Devices, USA).

### Statistical analysis

PCR was used as the main assay for assessing carriage of *S*. *pneumoniae*. However, for two mothers’ and four infants’ PCR results were missing so culture results were used for carriage. There was no positive culture that was PCR negative; there were five PCR positive and culture negative results. Therefore, nasopharyngeal carriage was defined as isolation of *S*. *pneumoniae* by PCR or by culture when the PCR result was missing. Continuous variables were log-transformed prior to analysis. Nasopharyngeal carriage was analyzed using a mixed effects logistic regression to account for correlation across time points. Mothers’ carriage and infants’ bacterial densities were also compared at each time point using Wilcoxon signed-ranks test. Mother and infant antibody concentrations were analyzed using Wilcoxon signed-ranks test at birth. A random-slope linear regression model with Kenward-Roger’s adjustment [26] was fitted to compare the infants’ antibody concentrations between carriers and non-carriers across time points and to assess the effect of maternal’ antibody concentrations on infants’ at birth. These effects were adjusted for mothers’ nasopharyngeal carriage and age, as well as infants’ gender, health facility of birth and infants’ antibiotics uptake. A joint model allowing for endogeneity as suggested by Skrondal and Rabe-Hesketh [27] was used to assess the effect of past infants’ nasopharyngeal carriage status on their future carriage status as well as the association between their antibody concentrations and carriage status. Finally, the effect of infants past nasopharyngeal carriage and antibodies concentrations on their future antibodies concentrations against *CbpA*, *PspA* and *rPly* was assessed using structural equation modeling [28]. Raw data is provided in S1 Table.

## Results

### Study population

One hundred and twenty pregnant women were enrolled into the study following written informed consent, 67 of these were seen at Brikama health centre while the rest were from the Fajikunda health centre (Table 1). There was no difference in mother’s age at delivery between health centres (median (IQR): 27 years (22–31) at Brikama health centre and 26 years (23–29) at Fajikunda health centre; p = 0.50). All study infants were full term babies; there was no difference in gender distribution between the health centres (p = 0.15). The median (IQR) birth weight was 3.1 kg (2.8–3.4) and 3.3 kg (3.0–3.5) in Brikama and Fajikunda health centres respectively, with some evidence of a difference in the birth weight between the health centres (p = 0.02) but there was no difference in the birth weight between boys and girls (p = 0.22).

**Table 1:** Characteristics of mothers and children at delivery or birth by health centre.

	Total (n = 120)	Brikama centre (n = 67)	Fajikunda centre (n = 53)	p
Mother’s age (years), median (IQR)	26 (22–30)	27 (22–31)	26 (23–29)	0.50 ^a^
Birth weight in Kg, median (IQR)	3.2 (3–3.5)	3.1 (2.8–3.4)	3.3 (3–3.5)	0.02 ^a^
Child gender, n (%)				
Male	59 (49.2)	29 (43.3)	30 (56.6)	0.15 ^b^
Female	61 (50.8)	38 (56.7)	23 (43.4)	
Mothers’ baseline antibody concentrations, median (IQR)				
Against *CbpA*	5790 (3987–10868)	6406 (4215–11461)	4946 (3340–9626)	0.04 ^a^
Against *PspA*	27373 (19126–59020)	32205 (21728–55839)	23620 (18450–60517)	0.19 ^a^
Against *rPly*	5666 (3504–10384)	4930 (3195–10210)	7623 (3922–11525)	0.09 ^a^
Infants’ baseline antibody concentrations, median (IQR)				
Against *CbpA*	6021 (4241–10919)	6357 (4410–11016)	5214 (3660–10697)	0.17 ^a^
Against *PspA*	5141 (3208–7813)	4957 (3107–7029)	5420 (3438–9997)	0.24 ^a^
Against *rPly*	6075 (3448–9713)	6771 (3343–9899)	5974 (3781–9680)	0.67 ^a^

^a^ Wilcoxon signed-ranks test.

^b^ Chi-square test.

### Concentrations of antibodies to CbpA, PspA and rPly in mothers and infants

**Fig 1:**
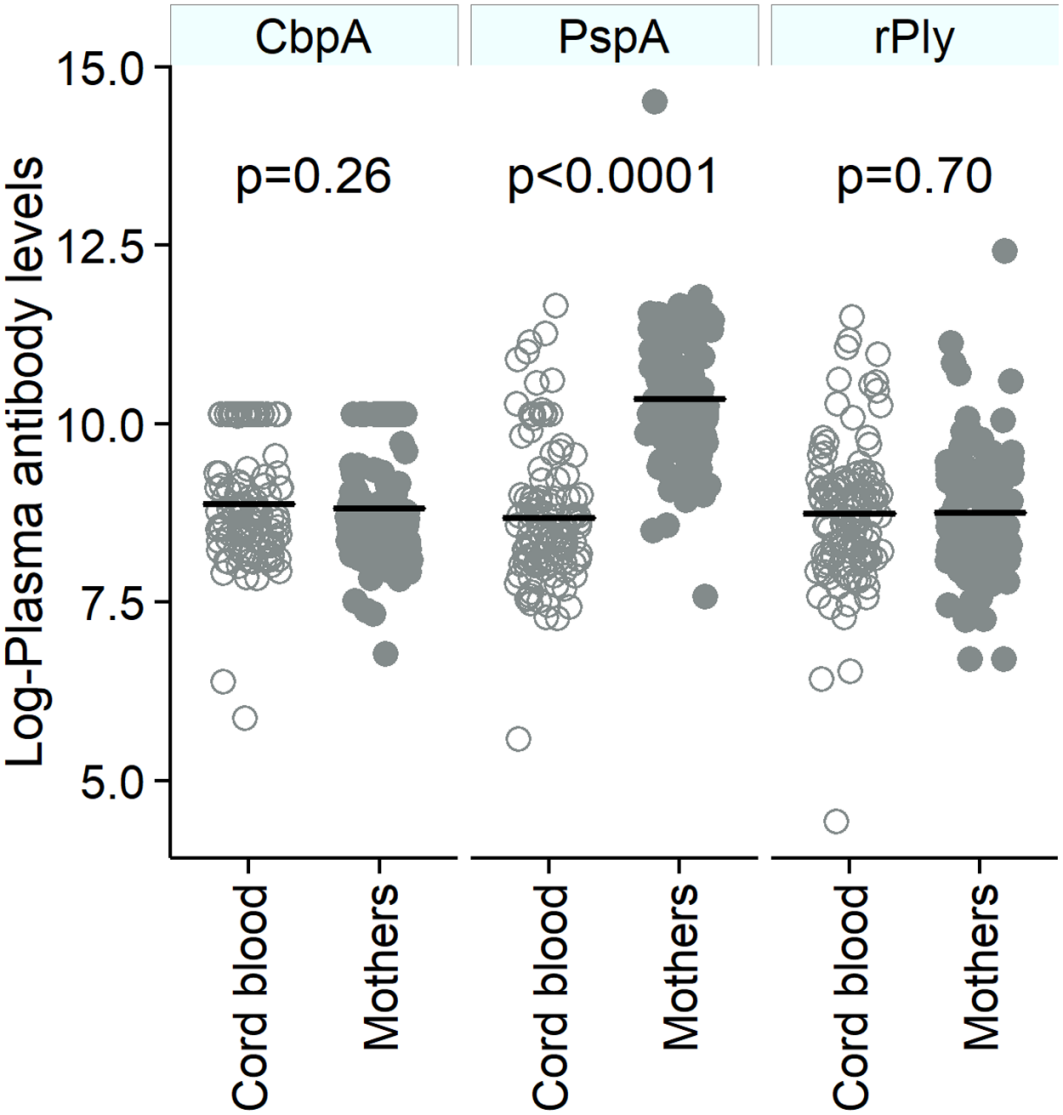
Concentration of antibodies against *CbpA*, *PspA* and *rPly* in mothers’ (closed circles) and cord blood (open circles) at birth. The horizontal black lines represent the average log-plasma antibody concentrations. The p values indicate the difference between the average antibody concentration for the specific pneumococcal antigen between mothers’ venous blood and infants’ cord blood at birth.

There was no difference in the concentrations of antibodies against the three protein antigens in the mothers or infants between the two health centres (Table 1). At birth, antibody concentrations against *CbpA* antigen in the mothers and in cord blood were comparable (p = 0.26; Fig 1) with a mean (SD) log-antibody concentration of 8.8 (0.8) and 8.9 (0.8) in mothers and infants respectively. Likewise, the antibody concentrations against *rPly* antigen in mothers (mean (SD): 8.8 (0.9)) and children (mean (SD): 8.7 (1.0)) were similar (p = 0.70). However, the antibody concentration against *PspA* protein antigen was higher in mothers compared to infants (mean (SD): 10.3 (0.9) vs. 8.7 (1.0); p<0.0001). There was strong evidence that the higher the mothers’ antibody concentrations against antigens *CbpA* and *rPly* were at delivery, the higher those antibodies’ concentrations were in the infant at birth (p < 0.001 for each antigen (Table 2). However, no association was found in the antibody concentration against *PspA* at birth between mother’s and infants’ (p = 0.91). In contrast to the situation at birth, there was no association between mothers’ antibody concentrations against *CbpA* at delivery and infants’ antibody concentrations against *CbpA* at four or eight weeks after birth (p = 0.97 and p = 0.57 respectively). As opposed to *CbpA*, the concentration of antibody against *PspA* in mothers at delivery was positively associated with infants’ antibody concentration against *PspA* at four weeks after birth (change per unit increase in mothers’ antibody concentration at delivery: 0.48 [95% CI: 0.31–0.65], p<0.0001) and eight weeks (change per unit increase in mothers’ antibody concentration at delivery: 0.40 [95% CI: 0.22–0.57], p<0.0001). In the case of *rPly*, there was strong positive evidence of an association between mothers’ antibody concentration against *rPly* at delivery and that of the infants at four and eight weeks after birth (p<0.0001 and p = 0.001 respectively). However, the strength of this association with respect to *rPly* decreased at four weeks after birth (change per unit increase in mothers’ antibody concentration at delivery: 0.55 [95% CI: 0.36–0.75], p<0.0001) and at eight weeks (change per unit increase in mothers’ antibody concentration at delivery: 0.32 [95% CI: 0.13–0.52], p = 0.001) compared to the change per unit increase at birth (0.89 [95% CI: 0.72–1.06]).

**Table 2:** Effects of mother’s log-antibody concentrations against *CbpA*, *PspA* and *rPly* at delivery on infant’s log-antibody concentrations and difference in infant’s antibody concentrations between carriers and non-carriers at birth, 4 and 8 weeks.

Main explanatory variables	Effect on infant’s log-antibody concentration against *CbpA*		Effect on infant’s log-antibody concentration against *PspA*		Effect on infant’s log-antibody concentration against *rPly*	
	Change (95% CI)	p	Change (95% CI)	p	Change (95% CI)	p
Mother’s log-antibody concentrations against corresponding antigen at delivery ^a^						
At birth	0.75 (0.56; 0.93)	<0.0001	0.01 (-0.16; 0.18)	0.91	0.89 (0.72; 1.06)	<0.0001
4 weeks	0.004 (-0.20; 0.20)	0.97	0.48 (0.31; 0.65)	<0.0001	0.55 (0.36; 0.75)	<0.0001
8 weeks	0.06 (-0.14; 0.26)	0.57	0.40 (0.22; 0.57)	<0.0001	0.32 (0.13; 0.52)	0.001
Infant’s log-antibody concentration against *CbpA*	-	-	0.27 (0.16; 0.38)	<0.0001	0.01 (-0.11; 0.13)	0.89
Infant’s log-antibody concentration against *PspA*	0.20 (0.10; 0.30)	<0.0001	-	-	0.13 (0.02; 0.24)	0.03
Infant’s log-antibody concentration against *rPly*	-0.01 (-0.11; 0.09)	0.81	0.12 (0.01; 0.22)	0.03	-	-
Infant’s carriage status (*ref*: Non-carrier)						
Carrier	-0.12 (-0.30; 0.05)	0.17	0.14 (-0.04; 0.33)	0.13	0.02 (-0.18; 0.21)	0.86
Mother’s carriage status (*ref*: Non-carrier)						
Carrier	-0.01 (-0.18; 0.17)	0.94	0.05 (-0.13; 0.24)	0.58	-0.07 (-0.26; 0.12)	0.48

^a^ Corresponding antigen is: *CbpA*, *PspA* and *rPly* if the outcome is infant’s log-antibody concentration against *CbpA*, *PspA* and *rPly*, respectively.

All reported effects were also adjusted for mother’s age, infant’s gender, infant’s uptake of antibiotics and birth health centre.

### Nasopharyngeal carriage in mothers and infants

**Fig 2:**
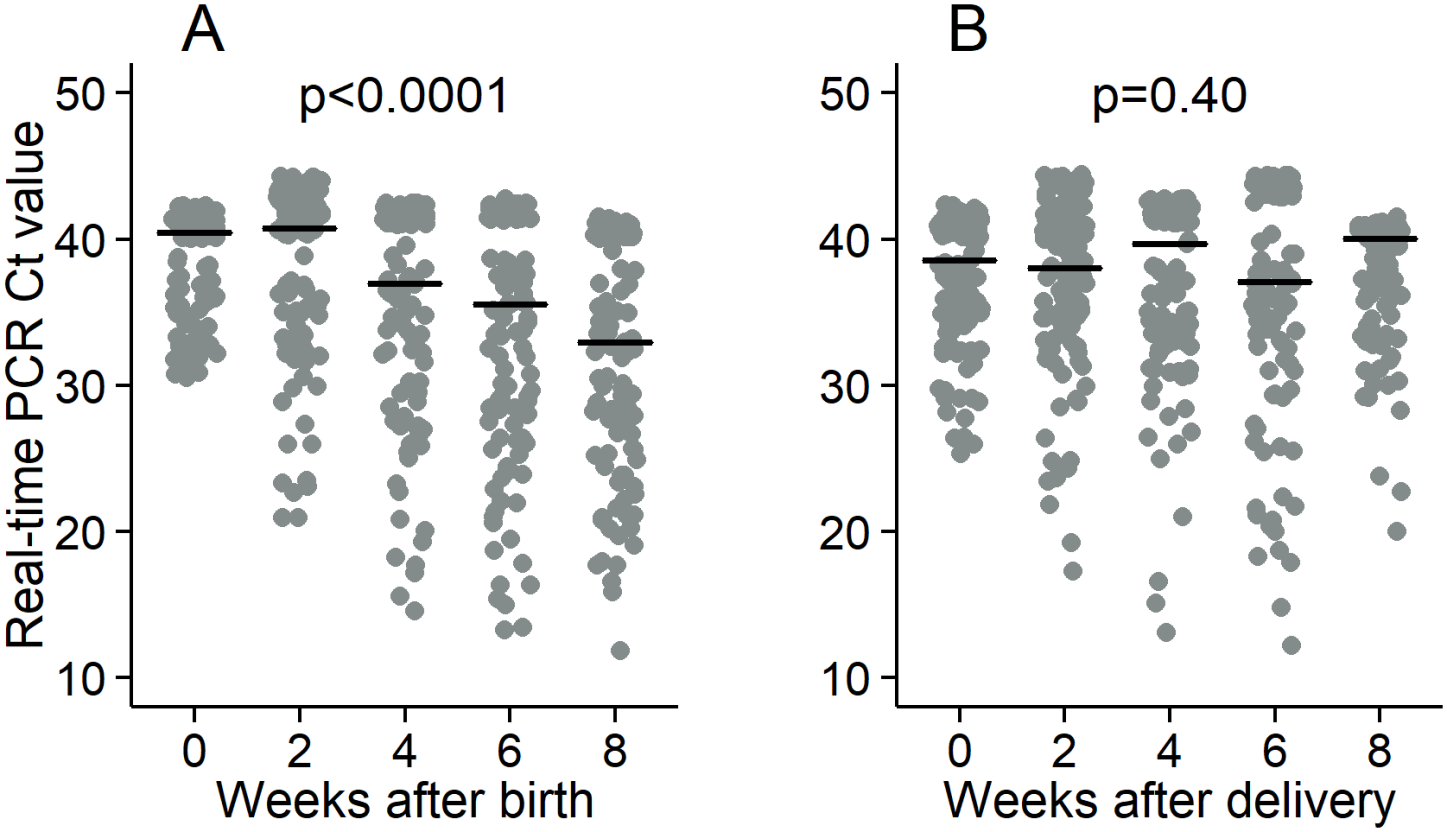
The density of *S*.*pneumoniae* in the nasopharynx in the first eight weeks after birth in infants (A) and the first eight weeks after delivery in mothers (B). The horizontal black lines represent the average Real-time Ct value, which is inversely correlated with the density. The p value indicates the evidence of variation of the density over the eight week period.

The nasopharyngeal carriage of *S*. *pneumoniae* in mothers and infants using either PCR alone or PCR and culture when PCR result was missing was similar (Table 3). The nasopharyngeal carriage was low in infants at birth as expected, stable over the first four weeks after delivery, increased at 6 weeks and decreased at 8 weeks (Table 3). In addition, there was a difference in pneumococcal colonization density across time points in infants (p<0.0001, Fig 2A). A contrasts analysis with Sidak’s multiple testing adjustment showed a difference in pneumococcal colonization density between two and four weeks (p = 0.002) but no difference between zero and two weeks (p = 0.84), between four and six weeks (p = 0.42) and between six and eight weeks (p = 0.53). As opposed to infants, mothers’ pneumococcal colonization density remained similar over the study period (p = 0.40, Fig 2B). Mothers’ pneumococcal colonization density at delivery were significantly higher than those of the infants at birth (p = 0.0004), but not at two weeks (p = 0.11). Pneumococcal colonization density were higher in infants compared to the mothers at four weeks (p = 0.009), six weeks (p = 0.002) and eight weeks (p<0.0001). Infants’ nasopharyngeal carriage four weeks earlier and their current carriage status were not associated (p = 0.28, Table 4). However, the odds of carriage in infants was 6.2 times (95% CI: 2.0–18.9) higher when their mothers were carriers within the same week than when they were not a carrier (p = 0.001). No association between antibiotics uptake and nasopharyngeal carriage in infants was found (p = 0.57).

**Table 3:** Nasopharyngeal carriage rate and 95% CI in mothers and infants at birth, 2, 4, 6 and 8 weeks.

Time after delivery	Risk (95% CI) in mothers		Risk (95% CI) in infants	
	PCR alone	PCR or culture ^a^	PCR alone	PCR or culture ^a^
At birth	53.3 (16.9–86.5)	53.3 (16.9–86.5)	35.4 (8.7–76.0)	34.7 (8.4–75.5)
2 weeks	57.57 (19.5–88.5)	57.7 (19.5–88.5)	41.8 (11.1–80.5)	41.7 (11.0–80.5)
4 weeks	51.6 (15.9–85.7)	51.6 (15.9–85.7)	57.9 (19.3–88.8)	57.9 (19.2–88.8)
6 weeks	61.8 (22.2–90.2)	61.7 (22.1–90.1)	68.3 (26.9–92.6)	67.7 (26.3–92.5)
8 weeks	45.3 (12.8–82.4)	45.3 (12.8–82.4)	69.9 (28.3–93.2)	69.3 (27.7–93.0)

^a^ Definition of nasopharyngeal carriage used in our study: isolation of *Streptococcus pneumoniae* by PCR or by culture when PCR result was not available.

**Table 4:** Dynamic of nasopharyngeal carriage and influence of concentration of antibodies against *CbpA*, *PspA* and *rPly* in infants.

Main explanatory variables ^a^	Effect on infant’s current nasopharyngeal carriage	
	OR (95% CI)	P
Infant’s carriage status four weeks earlier (*ref*: Non-carrier)		
Carrier	0.41 (0.08–2.09)	0.28
Infant’s current log-antibody concentration against *CbpA*	0.49 (0.26–0.92)	0.03
Infant’s current log-antibody concentration against *PspA*	1.00 (0.42–2.37)	0.99
Infant’s current log-antibody concentration against *rPly*	1.41 (0.72–2.75)	0.32
Mother’s current carriage status (*ref*: Non-carrier)		
Carrier	6.20 (2.03–18.9)	0.001
Infant’s current uptake of antibiotics (*ref*: No)		
Yes	1.71 (0.27–10.8)	0.57

^a^ Time-invariant variables such as infant’s gender, birth health centre, mother’s age, mother’s log-antibody concentrations against *CbpA*, *PspA* and *rPly* at delivery were also included in the models

### The relationship of the antibody concentrations to nasopharyngeal carriage in infants

The ability of antibodies against the pneumococcal proteins to protect against nasopharyngeal carriage was addressed by comparing the concentrations of those that carried with those that did not at the three time points that the antibodies were measured. This was analyzed using dynamic logistic regression with joint working models allowing for endogeneity of time-varying covariates (Table 4). Log-antibody concentrations against *CbpA*, *PspA* and *rPly* in infants were analysed using a linear dynamic panel data model (Table 4). A negative association between the current antibody concentration against *CbpA* antigen and nasopharyngeal carriage in infants (p = 0.03, Table 4) was found [OR (95% CI): 0.49 (0.26–0.92]. In contrast there was no evidence of association between infants antibody concentrations and their chance of carriage for *PspA* [OR (95% CI): 1.00 (0.42–2.37), p = 0.99] or *rPly* antibodies [OR (95% CI): 1.41 (0.72–2.75), p = 0.32].

The log-antibody concentration against *CbpA* was higher by 0.27 (95% CI: 0.01; 0.53, p = 0.04, Table 5) in infants who were carriers four weeks previously compared to those who were non-carriers. No association was found between infants’ nasopharyngeal carriage four weeks earlier and their current log-antibodies concentration against *PspA* (p = 0.22) or *rPly* (p = 0.70). There was a strong positive association between log-antibodies concentration against *rPly* in infants four weeks earlier and their log-antibodies concentration against *rPly* four weeks later (change per unit increase: 0.31 (95% CI: 0.09; 0.52), p = 0.005) but this association was not found for *CbpA* (p = 0.79) or *PspA* (p = 0.76). The results from all analyses presented above did not change when nasopharyngeal carriage in infants or mothers was defined using the PCR assay alone.

**Table 5:** Dynamic relationship between concentration of antibodies against *CbpA*, *PspA* and *rPly* and nasopharyngeal carriage in infants.

Main explanatory variables ^a^	Infant’s current log-antibody concentration against *CbpA*		Infant’s current log-antibody concentration against *PspA*		Infant’s current log-antibody concentration *rPly*	
	Effect (95% CI)	P	Effect (95% CI)	p	Effect (95% CI)	p
Infant’s carriage status four weeks earlier (*ref*: Non-carrier)						
Carrier	0.27 (0.01; 0.53)	0.04	-0.12 (-0.31; 0.07)	0.22	0.05 (-0.19; 0.28)	0.70
Infant’s log-antibody concentration against *CbpA* four weeks earlier	0.03 (-0.20; 0.26)	0.79	-0.07 (-0.24; 0.10)	0.39	0.24 (-0.45; 0.03)	0.02
Infant’s log-antibody concentration against *PspA* four weeks earlier	-0.24 (-0.38; -0.09)	0.001	-0.02 (-0.15; 0.11)	0.76	-0.02 (-0.15; 0.12)	0.82
Infant’s log-antibody concentration against *rPly* four weeks earlier	-0.17 (-0.42; 0.09)	0.20	-0.19(-0.36; -0.02)	0.03	0.31 (0.09; 0.52)	0.005
Mother’s current carriage status (*ref*: Non-carrier)						
Carrier	0.20 (-0.08; 0.48)	0.17	0.13 (-0.12; 0.39)	0.31	0.18 (-0.08; 0.48)	0.17
Infant’s current uptake of antibiotics (*ref*: No)						
Yes	0.22 (-0.22; 0.67)	0.32	-0.06 (-0.45; 0.32)	0.75	0.05 (-0.42; 0.52)	0.83

^a^ Time-invariant variables such as infant’s gender, birth health centre, mother’s age, mother’s log-antibody concentrations against *CbpA*, *PspA* and *rPly* at delivery were also included in the models.

## Discussion

This study evaluated nasopharyngeal colonization with *S*. *pneumoniae* and its relationship to concentrations of antibodies against three major pneumococcal protein antigens in the first two months of life, the period before the first dose of PCV was given, in two peri-urban communities in The Gambia. The proportion of infants who carried the pneumococcus as well as the bacterial density of *S*. *pneumoniae* in their nasopharynx increased progressively from birth to the age of eight weeks, while prevalence and density in the mothers remained the same. The concentrations of antibodies against the pneumococcal proteins in the cord blood were high and comparable to those in the mothers at birth. The concentrations of antibodies against *CbpA* and *rPly* declined with time while the opposite was the case for antibodies against *PspA*. Higher antibody concentrations to *CbpA*, but not *PspA* and *rPly*, were associated with reduced nasopharyngeal carriage of *S*. *pneumoniae* in infants.

The possibility of direct protection of young infants, who are at high risk of invasive pneumococcal disease, by the pneumococcal IgG antibodies acquired from maternal-fetal transfer has led to consideration of immunizing pregnant mothers. [29] A number of factors including hypergammaglobulinaemia, HIV/AIDs and malaria affect transplacental transfer of antibodies [30, 31]. However, understanding of the factors that influence mother-to-child transfer of antibodies against pneumococcal antigens is limited. Our data show that maternal antibodies are passed through the placenta to newborns in similar concentrations for CbpA and rPly but not for PspA antibodies which were lower in infants than in their mothers. Although protective protein antibody concentrations are yet to be determined, our findings imply that antibodies against these antigens that might be induced in mothers during pregnancy by vaccination will be passed on to the newborns. However there is a risk that high concentrations of these antibodies in infants could limit responses to these protein antigens if given as vaccines in early infancy, as shown for other vaccines such as tetanus toxoid and measles [32–34].

As expected, the proportion of infants from whom a pneumococcus was isolated from the nasopharynx was low at birth and increased with age. This pattern of nasopharyngeal carriage is similar to that observed in rural Gambia villages [35]. However, the proportion of infants that carried in the current study was lower than that recorded in the previous rural study [33, 36]. This could be due to the fact that living conditions in the peri-urban area where our study was conducted have fewer risk factors associated with carriage compared to the rural areas. It could also be due to the indirect effect on nasopharyngeal carriage of PCV that was introduced into the routine immunization programme of The Gambia three years before our study was conducted as deployment of PCVs in infants can be associated with an indirect effect against pneumococcal carriage [1,37,38].

The increase in the pneumococcal colonization density with age in the infants within the first 8 weeks of life is likely to be due to cumulative acquisition of pneumococci of the same or different serotypes with increasing exposure to the environment with time. The increasing proportions of children that carry *S*. *pneumoniae*, coupled with the increasing density with age are likely to contribute to the increased incidence of invasive pneumococcal diseases in early infancy. Indeed, *S*. *pneumoniae* has been shown to colonize the nasopharynx for several weeks without any diseases, but IPD may ensue under conditions that are associated with an increase in bacterial density in the nasopharynx [39,40]. A previous study in The Gambia found a decrease in bacterial density and carriage rate with age, but this applied to an older age group [41]. It is possible that the pneumococcal colonization density and carriage rate of the infants in our study would reach a maximum shortly after observations were concluded and then begin to decline as they acquired immunity following the recurrent exposures and carriage with time [42, 43].

There was evidence that the higher the concentration of antibodies against *CbpA* in early infancy, the lower the chance of carriage in the infants. We could not determine a concentration of antibody against *CbpA* that could be used as a marker of protection, due in part to the relatively small sample size of this study. The lack of evidence for reduction in the risk of carriage by antibodies against *PspA* and *rPly* is similar to other studies [24,44]. This could be due to the fact that these antigens do not exert their effect on nasopharyngeal carriage through antibodies but by T cells, as previously described [45]. There was tendency for a higher concentration of antibodies against *PspA* to be associated with a higher carriage perhaps because antibodies against *PspA* are not able to protect against carriage and are an indicator of a risk factor for exposure. [46].

In conclusion, our study found that the naturally induced antibodies against three pneumococcal protein antigens were transferred from mother to infant. The proportion of infants with nasopharyngeal carriage and bacterial density of *S*. *pneumoniae* increased with age within the first eight weeks of life. Higher concentrations of antibodies against *CbpA*, but not *PspA* and *rPly*, were associated with a reduced risk of nasopharyngeal carriage of *S*. *pneumoniae* in infants.

## Supporting information

S1 Table The raw data are available in the “Master data_clean_final.xls”. (XLS)
